# Heating as a rapid purification method for recovering correctly-folded thermotolerant VH and VHH domains

**DOI:** 10.1186/1472-6750-7-7

**Published:** 2007-01-26

**Authors:** Aurelien Olichon, Daniel Schweizer, Serge Muyldermans, Ario de Marco

**Affiliations:** 1 Cell Biology Unit, EMBL – Meyerhofstr. 1, D-69117, Heidelberg, Germany; 2 Scientific Core Facilities, EMBL – Meyerhofstr. 1, D-69117, Heidelberg, Germany; 3 Department of Cellular and Molecular Interactions, VIB, Vrije Unversiteit Brussel, Pleinlaan 2, B-1050, Brussel, Belgium; 4 IFOM-IEO Campus, Biochemistry Unit, via Adamello 16, I-20139, Milano, Italy

## Abstract

**Background:**

Recombinant antibodies from Camelidae (VHHs) are potentially useful tools for both basic research and biotechnological applications because of their small size, robustness, easy handling and possibility to refold after chemio-physical denaturation. Their heat tolerance is a particularly interesting feature because it has been recently related to both high yields during recombinant expression and selective purification of folded protein.

**Results:**

Purification of recombinant RE3 VHH by heat treatment yielded the same amount of antibody as purification by affinity chromatography and negligible differences were found in stability, secondary structure and functionality. Similar results were obtained using another class of thermotolerant proteins, the single domain VH scaffold, described by Jespers et al. [8]. However, thermosensitive VHs could not withstand the heat treatment and co-precipitated with the bacterial proteins. In both cases, the thermotolerant proteins unfolded during the treatment but promptly refolded when moved back to a compatible temperature.

**Conclusion:**

Heat treatment can simplify the purification protocol of thermotolerant proteins as well as remove any soluble aggregate. Since the re-folding capability after heat-induced denaturation was previously correlated to higher performance during recombinant expression, a unique heating step can be envisaged to screen constructs that can provide high yields of correctly-folded proteins.

## Background

There has been an increased interest in antibodies in recent years, both because of their clinical applications and their use in basic research [1]. Conventional antibodies (mono- and poly-clonal) present several shortcomings, such as their bulky structure, tedious and expensive preparation, limited opportunities to introduce mutations, and the immunogenic response they can induce when used in therapy. For these reasons, a techniques aimed at recombinant expression of antibodies selected from both immunized and naïve/synthetic libraries can be a convenient alternative [2].

The most common format for antibody recombinant expression is probably the single chain antibody (scFv), in which the heavy and light variable regions are linked together. Polybodies, with higher avidity for the antigen than single scFv molecules, can be obtained by varying the length of the linker or by connecting *via *a flexible hinge to an amphipathic helix [3,4]. ScFvs have been widely used to identify antigens in *in vitro *and *in vivo *experiments and to deliver active molecules against tumor markers in model animals [1].

The variable heavy (VHH) format is an alternative that exploits the particularity of the Camelidae immunogenic system. The animals belonging to this family possess, beside antibodies of conventional structure, also antibodies formed only by the heavy chain [5]. In this case all of the information for the specific recognition of the antigen is present in the heavy chain variable region. In contrast to the variable heavy chain of conventional antibodies (VH), which pairs with the light chain, the VHHs have evolved in the absence of such a counterpart. This results in a higher intrinsic stability of VHHs, in comparison to scFvs, when the recombinant antibodies are expressed in bacteria [6]. Since the paratope is mostly restricted to the extruding CDR3 region, VHHs preferentially bind antigens in small cavities otherwise not accessible to conventional and scFv antibodies. For example, the active sites of enzymes [7]. Furthermore, the ease in cloning and preparation of fusion constructs makes VHHs promising molecules for biotechnological applications like antibody-based microarrays and biosensors [5].

We wished to obtain stable recombinant antibodies for *in vitro *and *in vivo *studies. However, several of the recombinant antibodies expressed in bacteria are structurally unstable, signifying that good binders selected by phage display cannot be successfully used for practical applications. Therefore, VHHs were first selected by phage display from an immune library (Olichon and Surrey, unpublished data), and then an innovative approach based on thermotolerance was used to investigate their stability after recombinant expression.

Recently Jespers et al. [8] showed that thermotolerance of VH domains correlated with their yields of recombinant soluble protein. We were able to purify recombinant proteins fused to Archaea partners by heating *E. coli *lysates and to show that the treatment enabled, at the same time, to select monodispersed proteins because aggregated fusion proteins precipitated during the heat treatment [9]. VHHs are thermotolerant proteins and the results reported in this paper show that a heating step can be used to purify them and the VH domains, preserving their structure and monodispersity.

## Methods

### Subcloning, expression, and purification of VHH and VH constructs

The VH constructs C36, C47, and DP47a were a kind gift of Dr. Winter. The VHH RE3 was isolated by a phage display screen of a VHH immune library cloned into the pHEN4 phagemid ([10]; Olichon, unpublished data). The sequence corresponding to RE3 was subcloned into the pHEN6 vector and this transformed into XL Blue competent cells. Transformed cells were used to inoculate Terrific Brot. The culture was induced with 1 mM IPTG when the OD_600 _reached 0.5 and grown overnight at 28°C. The pellet corresponding to a 1 L culture was recovered by centrifugation and initially resuspended in 5 mL of TES buffer (0.2 M TrisHCl, pH 8.0, 0.5 mM EDTA, 0.5 M sucrose) before freezing in liquid nitrogen. It was then thawed on ice and 5 mL TES plus 5× EDTA-free protease inhibitor cocktail (Roche) were added. After 30 min incubation on ice, osmotic shock was induced by supplementing the cell suspension with 15 mL of 1:4 diluted TES buffer. After further 30 min incubation on ice, the cells were centrifuged 20 min at 30,000 g and the supernatant was recovered. NaCl and imidazole were added to a final concentration of 250 mM and 10 mM, respectively, and the fraction was purified by immobilized metal affinity chromatography (IMAC) using a HiTrap Chelating column (Amersham) and FPLC. The eluted fraction was desalted in 50 mM TrisHCl, pH 8.0, 50 mM NaCl and 10% glycerol and checked by SDS-PAGE. Its concentration was estimated after measurement of the absorbance at 280 nm.

The gel filtration experiments were performed using a Superpose 12 column (GE Healthcare) coupled to an FPLC system.

Heat purification of both RE3 and VHs was performed by heating the supernatants recovered after centrifugation of the bacterial periplasmic fraction and adding of PEG 6000 (10% final volume). Samples were heated 15 min at 70°C and thereafter cooled on ice for 20 min before pelleting the denatured proteins [11]. Thermotolerant proteins were recovered from the supernatant and analyzed by SDS-PAGE.

An ELISA test was performed according to [12] using both heat-treated and untreated RE3. A VHH directed against lysozyme was used as a control. VHHs were detected using an anti-His monoclonal antibody (Qiagen) and a secondary anti-mouse HRP conjugated antibody (Amersham).

### Fluorimetric and CD analyses

The fluorimetric assay proposed by Nominé et al. [13] has proved to be a reliable and simpler method than size exclusion chromatography for estimating the aggregation degree of proteins in solution [14]. It is the ratio between the adsorbance at 280 nm (light scattering due to soluble aggregates) and that at 340 nm (specific absorbance of the accessible aromatic groups). The measurements were performed using an AB2 Luminescence Spectrometer (Aminco Bowman). Samples were excited at 280 nm and the emission spectra between 260 and 400 nm were recorded.

Standard far-UV CD spectra of the recombinant antibodies were recorded at 20°C using a Jasco J-710 spectrophotometer and cuvettes of 1 mm pathlength. 15 runs were accumulated for each sample and three independent repeats were performed to confirm the results. The spectra of the unfolded proteins were determined after having increased the temperature to 95°C using a Peltier heater and those of the refolded proteins after a further incubation of 30 min at 20°C. Capped cuvettes were used to prevent sample evaporation.

The wavelength for which the difference of ellipticity value between the folded and unfolded states was maximum was selected for progressively heating the samples and to identify the melting temperature (Tm) of the protein. The temperature was increased at a rate of 30°C/hour from 15 to 95°C and the ellipticity values were recorded at every increase of 0.2°C.

## Results

### Purification of the RE3 VHH binder

The RE3 binder was first selected after panning a llama VHH phage display library using Ran-GTP, a protein involved in mitotic spindle assembly. It was sub-cloned in pHEN6 for expression in bacterial periplasm and showed both high affinity and specificity in enzyme-linked immunosorbant assay (ELISA) (Olichon, unpublished).

**Figure 1:**
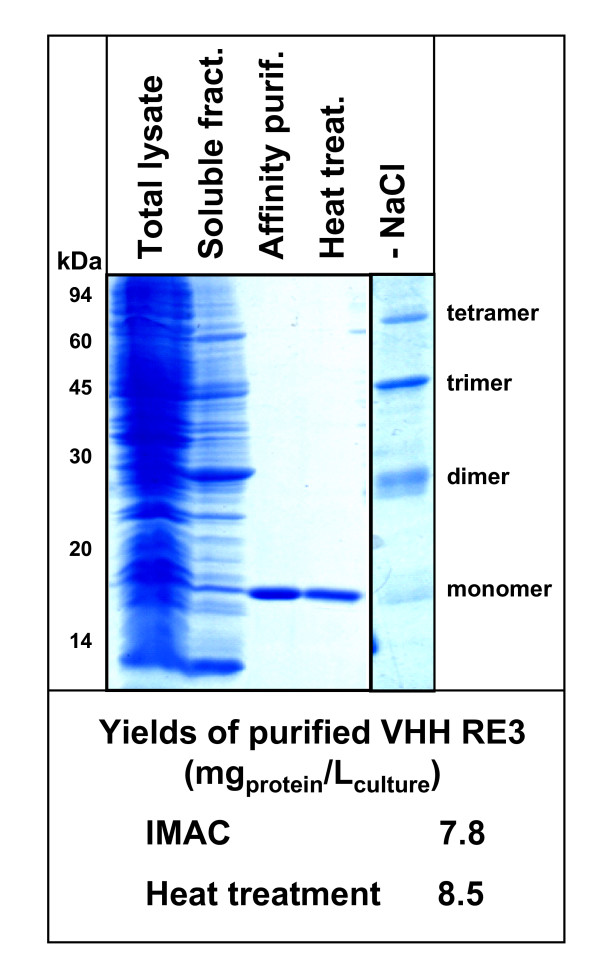
Purification of the RE3 VHH recombinant antibody. SDS PAGE and yields of RE3 purified by either IMAC or heat-treatment, in the presence or absence of 100 mM NaCl.

The first IMAC purifications resulted in a high yield of recombinant VHH. A stability test was performed using buffers at different pH and salt concentrations. The results indicated that the stability of the RE3 VHH was strongly salt dependent, since RE3 tends to polymerize and to form progressively large aggregates in the absence of NaCl. The polymerized forms were separated by SDS-PAGE in the absence of fresh DTT (Fig. 1). The addition of 100 mM NaCl was sufficient to prevent aggregation and to obtain monomeric VHH (Fig. 1). It can be speculated that NaCl prevents hydrophobic interactions favorable to the formation of intermolecular disulfide bonds. Using such conditions, 8 mg of VHH were purified from 1 L culture, and the low aggregation index indicated that the protein was monodispersed. In the presence of NaCl, the VHH did not aggregate even when kept at room temperature for several days (data not shown).

**Figure 2:**
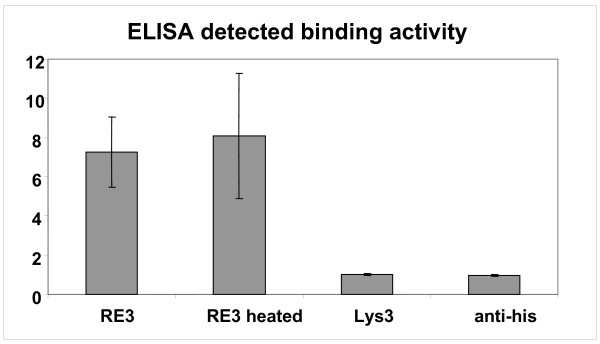
RE3 VHH binding capacity is not modified by heat treatment. ELISA was performed using samples of both heat-treated and control RE3, an unspecific VHH directed against lysozyme as a control, and in the presence of the only secondary antibody directed against the His-tag.

Protein aggregates tend to precipitate faster than monodispersed proteins when heated. Therefore, tolerance to heat treatments has been proposed as a way to selectively recover native proteins from a mixture in which aggregates are present [9]. As an alternative to IMAC, RE3 was purified by heating the supernatant recovered after osmotic shock of the bacterial cells for 15 min at 75°C. No significant differences in purity and yield were detected when compared to the affinity purified VHH (Fig. 1). In addition, the binding efficiency of RE3 measured by ELISA was not inhibited by the heat treatment (Fig. 2). Four further llama VHHs were recovered monodispersed in the soluble fraction after heat purification (data not shown), confirming that thermotolerance is not an exclusive feature of RE3 (See additional file 1).

**Figure 3:**
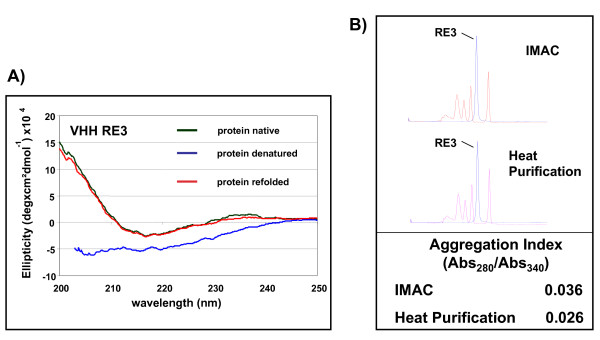
Stability of heat purified RE3 VHH. A) CD spectra of IMAC and heat-treatment purified RE3. B) Gel filtration profiles and aggregation indexes of the same samples. The protein peaks corresponding to the IMAC and heat-purified RE3 (blue) are compared to the profile of the molecular mass markers (red/pink).

Purified RE3 was used to understand what structural modifications happen during heat treatment. Figure 3 shows the CD spectra of the control protein (IMAC purified), the protein heated 15 min at 75°C, and the same heat-treated protein after 30 min on ice, indicating that VHH underwent total denaturation during the heat treatment but quickly refolded. Both gel filtration and the fluorimetric analysis confirmed its monodispersed structure (Fig. 3). The melting temperature for RE3 was determined using the CD and calculated at 65.3°C.

These results confirm that the antibody was expressed in a stable and native form in the bacterial periplasm and that its native structure was preserved during affinity purification and recovered after denaturation. However, its stability and monodispersity were strictly dependent on salt concentrations.

### VH antibodies can be purified by heat treatment

Binders belonging to a specific class of VH antibodies [8] have been shown to possess a large variability in their capability to refold after heat denaturation. In particular, the authors noticed a strong correlation between heat tolerance and yields of soluble antibodies expressed recombinantly in bacteria. We thought that it would be interesting to compare the heat tolerance features of RE3 with those of the described VHs.

**Figure 4:**
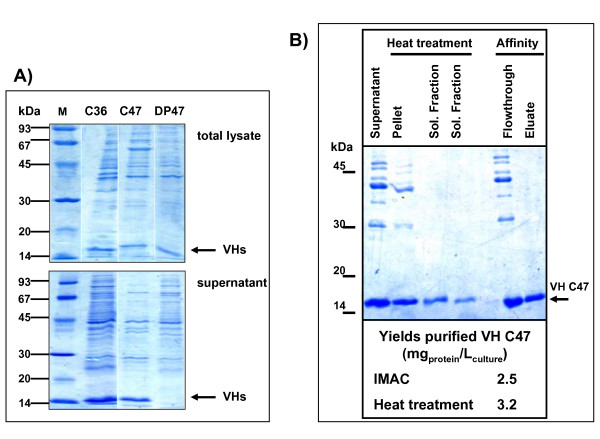
Purification of VH domain antibodies. A) Total lysates and soluble fractions obtained after periplasmic extraction of bacteria expressing the soluble VHs C36 and C47, as well as the insoluble VH DP47. B) Heat-treatment and affinity purification of C47. The supernatant obtained after periplasmic extraction was either heated 20 min at 70°C or loaded onto a cobalt-activated column for IMAC.

The heat tolerant VHs C36 and C47 as well as the heat sensitive DP47a were expressed and purified from bacteria periplasm. As expected, all three constructs were expressed and a recognizable band was detectable in each total lysate. However, only C36 and C47 were recovered soluble in the supernatant after centrifugation (Fig. 4A). Since phage displayed C47 refolded into an active structure after heat denaturation [8], purification by IMAC and by heat treatment were attempted. Similar yields of homogeneous C47 were recovered using both of the purification protocols (Fig. 4B).

**Figure 5:**
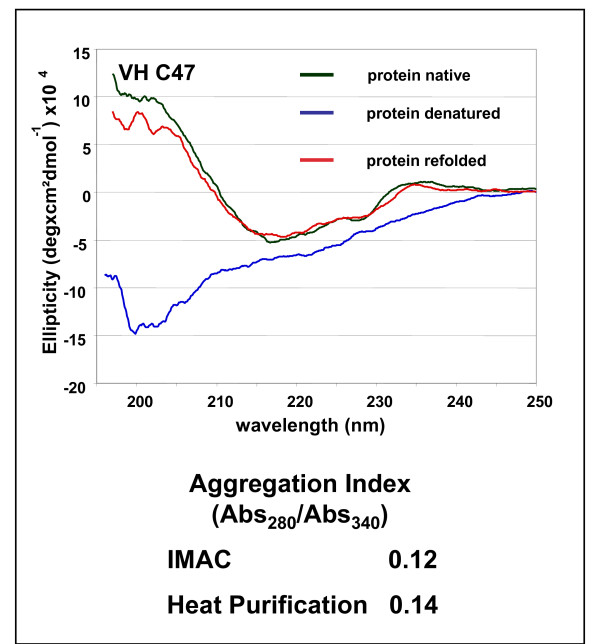
Heat tolerance of the VH soluble domain C47. The protein was denatured by a heat treatment (20 min × 70°C) and then left to recover 30 min on ice. CD spectra of native, heat-unfolded and refolded C47 were recovered and the aggregation indexes were measured at the fluorimeter.

The effect of the heat treatment was determined as described above for VHH. The secondary structure of the heat- and IMAC-purified C47 was analyzed by CD and the aggregation of the two samples evaluated at the fluorimeter. No significant difference was detectable (aggregation indexes of 0.12 and 0.14 for IMAC and heat-purified C47, respectively; CD data in Fig. 5), indicating that heat purification of thermotolerant proteins may represent an alternative to conventional affinity chromatography.

## Discussion and conclusion

The data show that both C47 and RE3 can withstand heat treatment. In particular, they are denatured but can correctly refold after heat denaturation and this feature enables their differential purification because the heating also denatures and precipitates thermosensitive bacterial proteins. The stability of RE3 seems to be dependent on the conditions of the buffer, and NaCl is necessary to prevent its progressive polymerization. Therefore, the robustness of RE3 under optimal conditions does not implicate that VHH is easy to handle and negative results obtained in some *in vitro *binding experiments might derive from its instability under the experimental conditions. Recently, a stable framework for the expression of VHH intrabodies has been proposed and a grafting strategy was suggested to transfer the CDR regions from any interesting but instable binder of the same HcAb subfamilly into such a structure [15]. It would be useful to challenge this framework for its buffer-tolerance as well, because the experience with RE3 clearly indicates that solubility and stability measured under optimal conditions are misleading whenever the application conditions significantly differ.

The original paper by G. Winter and co-workers indicates that only heat-denatured VHs that could correctly refold had structural features compatible with productive recombinant expression in bacteria [8]. Now we complete this analysis by showing that not only the selection but even the purification can be performed using a single heating step. Therefore, the reported experiments enable an interesting strategy for simplifying protein purification and combining it with quality control [16]. Such a strategy is based on the observation that not only the binding efficiency of RE3 was not inhibited by a heat treatment (15 min at 75°C) sufficient to denature the bacterial proteins, but other llama VHHs remained active after a heat treatment at 90°C [6] or were able to bind their substrate at 70°C [17]. Similarly, the binding capacity of VHs is not altered by a 10 min treatment at 80°C [8]. Furthermore, the results of the present work confirm those of the recent report showing that only monodispersed thermotolerant proteins can withstand a heat treatment [9]. It also confirms that the heat-purified antibodies had the same secondary structure and low aggregation value typical of their respective affinity purified antibodies. Therefore, we have demonstrated that heat treatment can be applied for the simultaneous purification and selection of native folded and active proteins belonging to thermotolerant classes such as VHs and VHHs (our study), as well as fusions with Archaea proteins [9,11].

Heat purification is not only a fast and inexpensive alternative to conventional chromatography but, when applicable, can also simplify the cloning strategy. Since no tag is needed for affinity purification, it is not necessary to produce tagged proteins that must be buffer exchanged, digested and re-purified before, for instance, being used in crystallography. The efficiency of heat purification with regard to the possibility to eliminate the endotoxins from bacterial recombinant protein preparations will be the object of specifically dedicated experiments.

It seems that thermotolerance, defined as the capability to refold after heat-denaturation, is sufficient for selective heat-dependent protein recovery. However, whilst thermoresistance is a very peculiar feature of extremophiles, thermotolerance can be identified even in proteins expressed in thermosensitive organisms, as with VHs and VHHs. We expect that other classes of thermotolerant proteins will be identified or their thermotolerance specifically improved by the addition of suitable compatible osmolytes [18].

## Competing interests

The author(s) declare that they have no competing interests.

## Authors' contributions

AO selected and produced the VHH antibody and participated in the manuscript writing. DS participated in the antibody purification and performed the biophysical assays. SM participated in the design of the phage display library. AdM conceived the experiments, performed part of the heat-purification experiments and drafted the manuscript. All the authors read and approved the final manuscript.

## Supplementary Material

Additional File 1 Seq_VHHs. A PDF File containing the sequences of the 5 VHHs used for the heat-dependent purification experiment. Click here for file
