# Supplementary material for: Heating as a rapid purification method for recovering correctly-folded thermotolerant VH and VHH domains
Source: BMC Biotechnol. 2007 Jan 26;7:7. doi: 10.1186/1472-6750-7-7 (PMC1790891; doi:10.1186/1472-6750-7-7)
Supplement: Additional File 1 — Seq_VHHs. A PDF File containing the sequences of the 5 VHHs used for the heat-dependent purification experiment. [file 1472-6750-7-7-S1.pdf]

Supplementary Figure 1. Alignment of the sequences corresponding to the 5 VHH antibodies used for the heat purification

```
seq1            MAEVQLQASGGGFVQAGGSLRLSCAASGR-TFSSYTMGWFRQAPGKEREFVAAISRSGGI 59
seq4            MAEVQLQASGGGLVQAGGSLRLSCAASGR-TDRRHAMGWFRQAPGKEREFVAGISRNGGT 59
seq2            MAEVQLQASGGGLVQAGGSLRLSCAASGWSGRSDYLIAWFRQARGKEREFVAAITWSGVN 60
seq3            MADVQLQASGGGLVQAGGSLTLSCAASER-LVSWFGMAWFRQAPEKEREFVASVLGSGS- 58
seq5            MADVQLQASGGGLVQPGGSLRLSCAASGF-RFSSYGMGWFRQASGKEREGVAAIRWDGGY 59
                **:*********:**.**** ******       . :.*****  **** **.:  .*

seq1            ASYSDSAKGRFTISRDNAKNTVYLQMNTLKPEDTATYYCAADPAYMTSTWYKRPYEYDYW 119
seq4            TYYTESVKGRFTISRDNAKNTVYLQMNMLKSEDTAVYYCNVK----YPGWLGR--NNEYW 113
seq2            LWYADSVKGRFTISRDTAN-TVYLEMNSMKPEDTAVYYCAADGPRGSGSRVYAG-RYDYW 118
seq3            ADYGDSVKGRFTISRDNARSTIYLQMNSLKPEDTAVYYCAAG--LGQGRAIST---YRYW 113
seq5            TRYADSVKGRFTASKDNAKNTMYLRMNNLKPEDTAVYFCGSARTPYDSLVTMSPSTIASW 119
                  * :*.***** *:*.*. *:**.** :*.****.*:*                     *

seq1            GQGTQVTVSSRGR 131
seq4            SQGTQVTVSSRGR 125
seq2            GQGTQVTVSSRGR 131
seq3            GPGDPVTVSSRGR 126
seq5            GQGTQVTVSSRGR 132
```
